# ﻿Reinstatement of *Memecylonelegantulum* (Melastomataceae) and recircumscription of *Memecylonrostratum*, two species endemic to Sri Lanka

**DOI:** 10.3897/phytokeys.259.146534

**Published:** 2025-06-19

**Authors:** Amila Perera, Himesh Jayasinghe, Bhathiya Gopallawa, Isuru Madawala, Nimal Gunatilleke, Nalaka Geekiyanage

**Affiliations:** 1 Postgraduate Programme, Faculty of Agriculture, Rajarata University of Sri Lanka, Anuradhapura 50000, Sri Lanka; 2 Dilmah Ceylon Tea Company PLC, No 111, Negombo Road, Peliyagoda, 11830, Sri Lanka; 3 National Institute of Fundamental Studies, Hantane Road, Kandy 20000, Sri Lanka; 4 Postgraduate Institute of Science, University of Peradeniya, Peradeniya 24000, Sri Lanka; 5 Agriculture Publication Unit, Department of Agriculture, Gannoruwa, Peradeniya 20400, Sri Lanka; 6 Department of Botany, Faulty of Science, University of Peradeniya, Peradeniya 24000, Sri Lanka; 7 Department of Plant Sciences, Faculty of Agriculture, Rajarata University of Sri Lanka, Anuradhapura 50000, Sri Lanka

**Keywords:** Endemic, low land wet zone, Sri Lanka, typification

## Abstract

*Memecylonelegantulum* Thwaites, a heterotypic synonym of *M.rostratum* Thwaites, is re-instated based on recent collections and field observations. The two species differ mainly in their habit, inflorescence structure, floral morphology and lamina morphology. A lectotype and an epitype are designated for *Memecylonelegantulum*, and a lectotype designated for *M.rostratum*. Both species are confined to the mixed dipterocarp rainforests of Sri Lanka’s perhumid south-western ‘wet zone’. *Memecylonelegantulum* appears to be restricted to a relatively small range in Ratnapura district, while *M.rostratum* has a wider distribution in wet zone.

## ﻿Introduction

The genus *Memecylon* L. is the largest genus of the subfamily Olisbeoideae, one of the three subfamilies in Melastomataceae (Christenhusz et al. 2018; Penneys et al. 2022). This genus consists of shrubs and trees usually with pinnately veined leaves of opposite arrangement and comparatively small flowers (Bremer 1979). While Renner et al. (2022) considered the genus to comprise more than 350 species, POWO (2024) estimates it to be substantially richer, and new species continue to be reported in substantial numbers (Stone 2022, 2023). The genus is distributed across the Old-World tropics, from Africa to Australia, including South and Southeast Asia (Maxwell 1980; Bremer 1987; POWO 2024), with many of the species exhibiting restricted distributions (Stone 2012; POWO 2024).

Having originated in Africa in the Eocene, *Memecylon* appears to have dispersed to South Asia through long-distance dispersal events (Amarasinghe et al. 2021). Being a continental island that was frequently connected to India by the Palk Isthmus during sea ice ages since the Miocene (Pethiyagoda and Sudasinghe 2021), however, the lineages of *Memecylon* in Sri Lanka are closely related to those of India. The latest revision of the genus for Sri Lanka (Bremer 1987) recognized 32 species (28 of them being endemic), distributed throughout the island, from the strongly seasonal arid zone to the perhumid wet zone, ranging from near sea level to the highest peaks over 2000 m in elevation. Recent discoveries of range extensions of some species from South India (Viswanathan and Rajendran 1993; Viswanathan 1995; Murugan and Manickam 2001; Kumar et al. 2004; Rajendraprasad et al. 2006; Ayyappan et al. 2012; Sivu et al. 2012; Udhayavani and Ramachandran. 2013), however, have reduced the number of endemics, calling for a critical review of earlier literature. The latest checklist (Wijesundara et al. 2020) considered 26 of the 32 species are endemic to Sri Lanka. Studies of *Memecylon* in Sri Lanka pre-date Linnaeus, who coined the generic name for two species collected from the island, by Paul Hermann in 1670s (Linnaeus 1753). Trimen (1894) considered *Memecylon* to be ‘one of the most difficult genera in Sri Lankan flora’, adding that the genus probably contained additional species in the island. Bremer (1979) too, mentioned that the material available to him was insufficient to fully understand the species already described and hence, to describe new species. Due to the lack of specimens and the poor condition of material available at the time, Bremer (1979) provisionally synonymized *Memecylonelegantulum* Thwaites under *Memecylonrostratum* Thwaites, retaining this synonymy also in his revision of the genus (Bremer 1987).

Recent field work in Sri Lanka’s wet zone has served to improve our understanding of the distribution and morphology of *M.rostratum* and its heterotypic synonym, *M.elegantulum*. It is clear from the material now at hand that *M.elegantulum* is a distinct species. We therefore reinstate this name, stabilizing its identity through the designation of a lectotype and an epitype. In this article, we provide diagnoses, descriptions and illustrations of both species.

## ﻿Materials and methods

Fieldwork was conducted during 2023 and 2024. Non-typical *Memecylonrostratum* was first encountered in Walankanda Forest Reserve in the Sinharaja Forest complex, in January 2023, during a floristics survey under Endane Biodiversity Corridor Project to understand the compositional variation of threatened flora along an elevation gradient. While georeferencing other species, special attention was paid to search for the taxon also in nearby forests. Throughout this period, data on both typical and non-typical *Memecylonrostratum* were gathered, including distributional range, morphological variability, and associated microhabitats. See Jayasinghe et al. (2022) for methodology associated with field collections, photography and measurements. Specimens collected were deposited in the National Herbarium, Peradeniya (PDA); abbreviations follow Thiers (2024). Additional specimens were examined in the collections of PDA, while specimens deposited in overseas herbaria (E, L, M, P, BR, US) were examined via JStor and the online resources available through those herbaria. Nomenclature follows the Shenzhen code (Turland et al. 2018), while author abbreviations and publication conventions follow IPNI (2024).

## ﻿Taxonomic treatment

### ﻿ Memecylon elegantulum

Taxon classificationPlantaeMyrtalesMelastomataceae

Thwaites, Enum. Pl. Zeyl. 112 (1859); Trimen, Handb. Fl. Ceylon, 2: 214 (1894)

1A969E79-CA66-56B2-AA50-67617216F144

Figs 1 , 2

Memecylon rostratum auct. *non* Thwaites, K.Bremer, Opera. Bot. 50: 21 (1979), *p.p.*; K.Bremer in Dassan., Revis. Handb. Fl. Ceylon 6:224 (1987), *p.p.*

#### Type.

• Sri Lanka *n.l.*, *n.d.*, *n.coll.*, C.P. 2684 (lectotype: third branch from the left of PDA [PDA00002924!], designated here); Samanala Watta side of Pettigala Forest Reserve, 17 iv 2023, *H.Jayasinghe et al*. *HDJ 2097* (epitype: PDA00109496, designated here).

**Figure 1.**
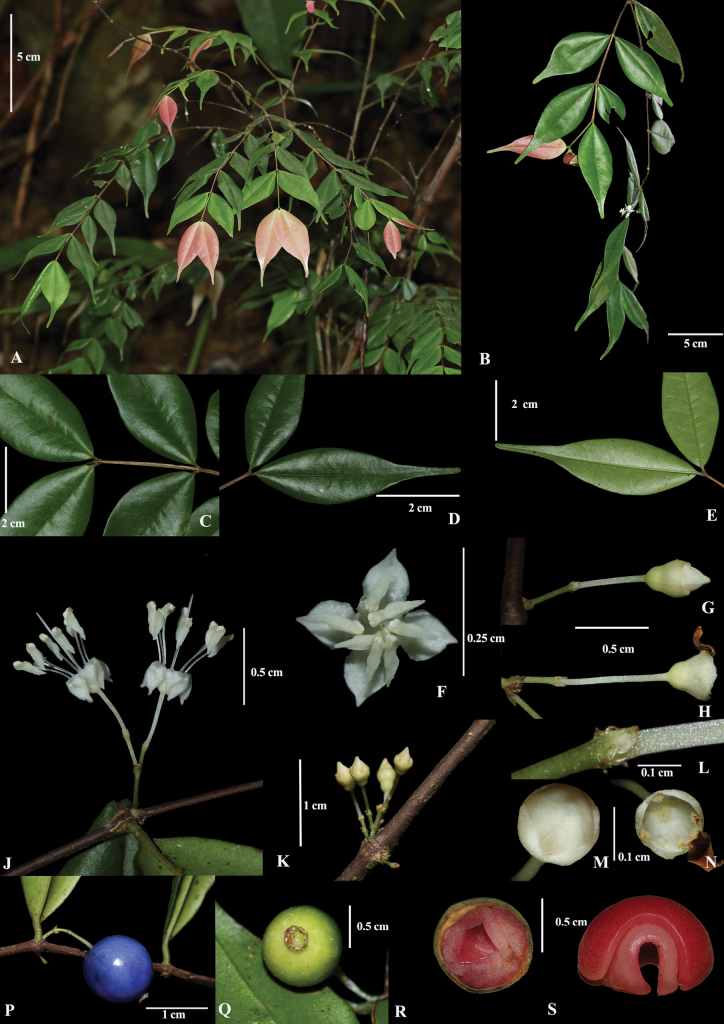
*Memecylonelegantulum* Thwaites; **A.** Habit; **B.** Branch with an inflorescence; **C.** Young stem; **D.** Lamina upper side; **E.** Lamina under side; **F.** Dorsal view of flower; **G.** Inflorescence with a single flower bud; **H.** Inflorescence with two flowers after anthesis; **J.** Lateral view of flowers; **K.** Two inflorescences arising from a single node; **L.** Bracts; **M.** Dorsal view of flower bud; **N.** Dorsal view of epigynous chamber after anthesis; **P.** Lateral view of partially ripe fruit; **Q.** Dorsal view of immature fruit; **R.** Longitudinal section of a fruit; **S.** Wrinkled cotyledons.

#### Description.

*Memecylonelegantulum* is distinguished from *M.rostratum* upto 2 m tall (vs treelet, to 7 m); having flush leaves deep purple-red to light pink (vs whitish green); branched, pedicellate inflorescence with 1–2 flowers (vs unbranched inflorescence with 6–9 capitate flowers); petals in bud conical with an apiculate tip (vs obtuse tip); anthers and anther connectives white (vs purplish-blue); straight anther connective without a gland (vs arched anther connective, with a prominent red gland); and fruit hanging, pale green in immature stage (vs erect, white).

**Figure 2.**
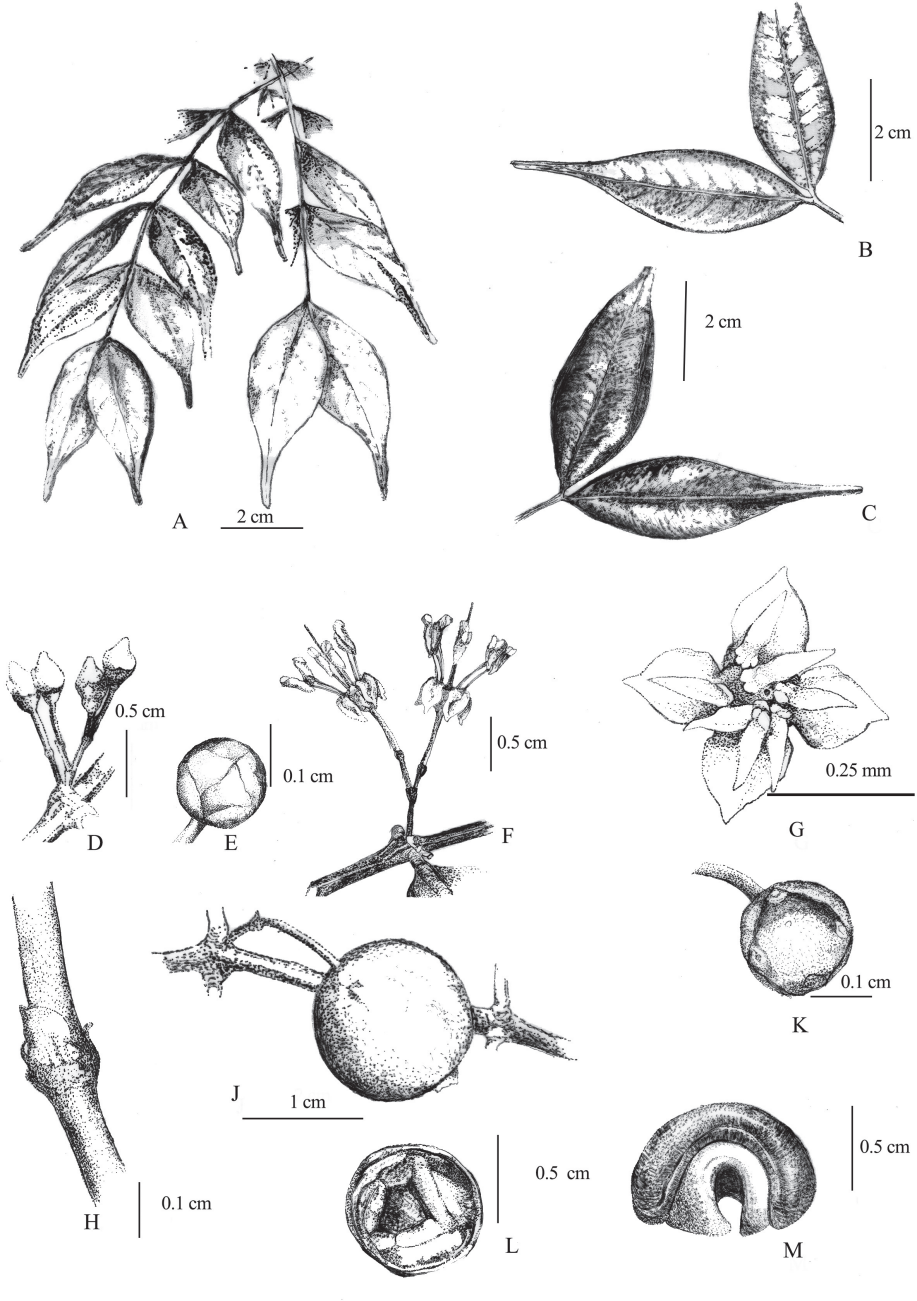
Line drawing of *Memecylonelegantulum* Thwaites; **A.** Branch; **B.** Lamina under side; **C.** Lamina upper side; **D.** Two inflorescences arising from a single node; **E.** Dorsal view of the flower bud; **F.** Lateral view of flowers; **G.** Dorsal view of flower, G- inflorescence with a single flower bud, **H.** Bracts; **J.** Lateral view of partially ripe fruit; **K.** Dorsal view of epigynous chamber after anthesis; **L.** Longitudinal section of a fruit; **M.** Wrinkled cotyledons.

Shrubs or treelets up to 2 m height; outer bark shallowly and longitudinally striate; young branchlets obscurely quadrangular, becoming terete with age; flush leaves deep purple-red to light pink; internode distance 18–25 mm. ***Leaves*** green above, much paler below, lustrous on both sides in live state, greenish-brown in dried state; petiole 1–2 mm long; lamina subcoriaceous, broadly to (rarely) narrowly elliptic, 40–65 × 15–20 mm, caudate to acuminate, obtuse at apex, narrowly obtuse to cuneate at base, margins slightly revolute toward base, slightly thickened; midrib slightly grooved adaxially, obscurely raised abaxially; lateral veins 7–9 pairs, with a few intermediaries, straight throughout, unicolorous in live state, venation visible on both sides in dried state; intramarginal vein 0.3–1 mm from the margin. ***Inflorescence*** 1 (–2) per node, axillary on lower leaf nodes or rarely below the existing leaf nodes; main axis of the peduncle 2.5–3.5 mm long, filamentous, quadrangular, pale yellowish green, topped by (1–) 2 capitate secondary axils, surrounded by minute bracts at the joint; secondary axils 2–3 mm long, filamentous, cylindrical, pale greenish white, topped by 2, minute, whitish bracts, holding a single flower. ***Flowers*** pedicel 4.5–5 mm long, white; hypantho-calyx broadly pyriform to infundibuliform, 1.7–1.9 mm long, 2.3–2.5 mm wide, outside smooth, white, sometimes with a bluish tinge at apex; calyx lobes 4, minute, obtuse to acute at apex; epigynous chamber smooth, without any furrows; exposed petals conical with a pointed apex in bud, white at anthesis, reflexed, 2.4–2.6 mm long, 1.8–2.1 mm wide; filaments 3.4–3.7 mm long, white; anther connective straight, 2.2–2.7 mm long, 0.6–0.7 mm wide, white; without a gland; anthers white; style 5.9–6.1 mm, white. ***Fruits*** 1–2 per inflorescence with an elongate, hanging pedicel up to 7–8 mm; subglobose, 9.5–11 × 7.5–9 mm diameter, topped by a persistent calycinal crown; surface smooth, yellowish green during immature stage, purplish blue at partial maturity, then turning blackish purple at maturity; cotyledons wrinkled.

#### Distribution and habitat.

**Figure 3.**
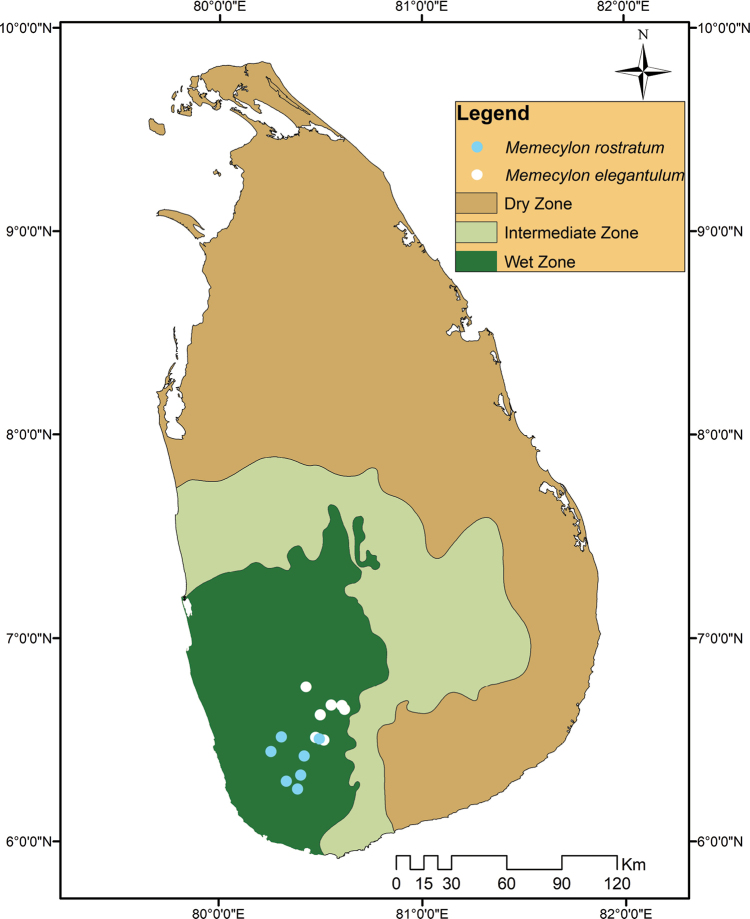
Distribution map *Memecylonelegantulum* Thwaites and *M.rostratum* Thwaites.

Lowland rainforests of Sri Lanka, northwards to Sinharaja Forest in the elevation range 250–800 m (Fig. 3). It usually occurs at the lower level of the rainforest understorey, on ridges and as well as in valleys.

#### Phenology.

Flowering and fruiting were recorded twice a year, from February to April and from August to November.

#### Notes.

In the protologue of *Memecylonelegantulum*, Thwaites (1859) briefly described its inflorescence, flower and the fruit. All the morphological features and distribution details he provided, except the description of the pedicel, are consistent with our own observations. Thwaites mentioned, however, that the pedicel was ‘as half as long as the calyx [*calyce dimidio longioribus*], while the taxon described here has pedicels approximately three times long as the calyx. Later, Trimen (1894) described the flower as sessile, which was repeated by Alston (1931). After a thorough search in herbaria, we encountered four syntypes. Although Trimen (1894) and Alston (1931) detailed the flower and the fruit, the syntypes we examined contained neither flowers nor fruits except for the crushed parts of a fruit in the pocket of BM000944509! and an immature fruit in the pocket of PDA00002922!. Although Trimen (1894), in his enumeration, mentioned that he had only scant material of this taxon, we were unable to locate any material other than the mentioned syntypes prior to Trimen’s time.

The drawing made from C.P. 2684 by H. de Alwis, Thwaites’ draftsman (Pethiyagoda 2007) curated at PDA, is a leafy branch with a single hanging fruit, which is bluish purple. This species has the longest pedicel (relative to the length of the hypanthocalyx) among the Sri Lankan species of *Memecylon*, while having a unique inflorescence architecture. Given that Thwaites noted that inflorescence was ‘sparsely racemose’ [*parce ramosis*], it is possible that he was misled by this feature, thinking that the pedicel was a secondary axis of the inflorescence. The second branch from the left of PDA00002924! has two broken primary axils of inflorescences, highlighting its filamentous nature. The third branch from the left of PDA00002924! has a single broken inflorescence axis, including a part of the secondary axis. This inflorescence section features the bracts at the inflorescence branching.

Since little Sri Lankan material was available to him, Bremer (1979) provisionally synonymized *Memecylonelegantulum* under *M.rostratum*. Bremer (1979) lectotypified the name as ‘C.P. 2684 in PDA’ while considering C.P. 2684 in BM & K to be iso-lectotypes. We note, however, that there are two sheets labelled C.P. 2684 in PDA, both of poor quality. As detailed by Jayasinghe et al. (2022), a single C.P. number often included multiple gatherings. PDA00002922! has an indistinct pencil notation about the gathering information (possibly ‘Gilimale, March 1853’) while PDA00002924! lacks any such information. This suggests that these specimens may have been the result of multiple gatherings. It is important, however, that type specimens be from a single gathering (see Article 8.2, footnote 1). Here under Art. 9.3, newly lectotypify the name, selecting the specimen in the best condition. Since the syntpes represent multiple gatherings, they are retained as such, without considering them for iso-lectotypification. Given that all the syntypes currently lack flowers as well as a complete peduncle, they only partially represent the taxon. Hence, we designate a flowering specimen as an epitype (Article 9.9).

#### Specimens examined.

**Sri Lanka**: • **Ratnapura District**: Kalawana, 30 iv 1970, *N.Balakrishnan NBK 315* (PDA, US02955738); Approximately 2 miles from Rassagala, 09 xi 1975, *S.H.Sohmer & S.Waas 10491* (PDA); • Bambarabotuwa Forest Reserve, 15 v 2018, *M.Gunathilake, N.Gunawardena & A.Sumanadasa NBS/2018/BAM/071* (PDA); • ibid., 14 v 2018, *M.Gunathilake, N.Gunawardena & A.Sumanadasa NBS/2018/BAM/023* (PDA); • ibid., *NBS/2018/BAM/013* (PDA); • ibid., 06 xi 2018, *B.Gopallawa & S.Gamage BAM 337* (PDA); • ibid., *BAM 203* (PDA); Botiyagala, Gilimale-Erathna forest, 21 viii 1993, *A.H.M.Jayasuriya & B.W.M.Wijesinghe 7478* (PDA); • Gilimale, Gilimale-Erathna forest, 4 vii 1993, *A.H.M.Jayasuriya & B.W.M.Wijesinghe 7415* (PDA); • ibid., 27 iii 2024, *H.Jayasinghe & Samarasinghe HDJ 2922* (PDA); • Dotalugala forest, 27 viii 1976, *S.Waas 1831* (PDA, L2545519, E01411687); • Massenna forest reserve, above Rassagala estate, 25 x 1993, *A.H.M.Jayasuriya & B.W.M.Wijesinghe 7644* (PDA); • Kiribathgala forest reserve, 06 iv 2024, *H.Jayasinghe, D.Samarasinghe & S.Kanishka HDJ 2961* (PDA); • Walankanda, 19 i 2023, *H.Jayasinghe, A,Perera, I.Madawala HDJ 1947* (PDA); • Delwala, 27 i 2023, *H.Jayasinghe, A. Perera, I. Madawala HDJ 1956* (PDA) • **Unknown localities**: *s.l., s.d., s.coll., s.n*., C.P. 2684 (PDA00002922; remaining specimens other than the lectotype of PDA00002924; BM000944509, K000859185).

### ﻿ Memecylon rostratum

Taxon classificationPlantaeMyrtalesMelastomataceae

Thwaites, Enum. Pl. Zeyl. 111 (1859); Trimen, Handb. Fl. Ceylon, 2: 218 (1894); K.Bremer, Opera. Bot. 50: 21 (1979), p.p.; K.Bremer in Dassan., Revis. Handb. Fl. Ceylon 6:224 (1987), p.p.

2E61B163-4AB7-5C81-BE35-79E96AE2A22C

Figs 4 , 5

#### Type. •

Sri Lanka *n.l.*, *n.d.*, *n.coll.*, C.P. 1560 (lectotype: the largest branch with flowers [second from the left] of PDA [PDA00002923!], designated here)

**Figure 4.**
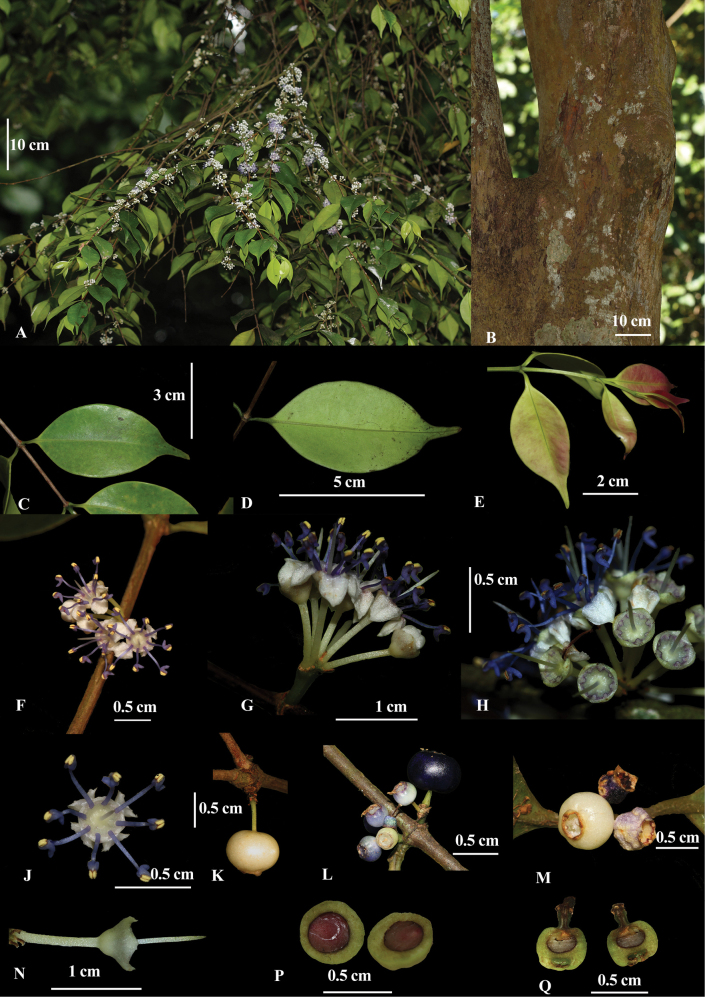
*Memecylonrostratum* Thwaites; **A.** Habit; **B.** Outer bark; **C.** Lamina upper side; **D.** Lamina under side; **E.** Flush leaves; **F.** Dorsal view of the inflorescence; **G.** Lateral view of the inflorescence; **H.** Dorsal view of epigynous chamber after anthesis; **J.** Dorsal view of the flower; **K.** Partially mature fruit; **L.** Immature fruits; **M.** Partially mature fruit and undeveloped fruits; **N.** Lateral view of flower immediately after anthesis; **P.** Cross-section of a partially mature fruit; **Q.** Longitudinal section of a partially mature fruit.

#### Description.

A small tree, to 8 m tall; outer bark smooth, not longitudinally striate, yellowish brown; young branchlets terete; flush leaves pale yellowish green, sometimes with a red tinge; internode distance 15–30 mm. ***Leaves*** green above, paler below, lustrous on both sides in live state, brown on upper side and yellow-brown on underside in dried state; petiole 2–5 mm long; lamina subcoriaceous, elliptic to ovate 40–70 × 25–38 mm, caudate to acuminate, obtuse to rounded at extreme apex, broadly to narrowly cuneate at base, margins flat, rarely slightly revolute towards base; midrib adaxially slightly grooved, abaxially obscurely raised; lateral veins 7–9 pairs, invisible in live state, hardly visible abaxially in dried state; intramarginal vein 0.3–0.5 mm from the margin. ***Inflorescence*** 1 (–2) per node, axillary, mainly on nodes below the existing leaves and extending in to the lower leaf nodes; peduncle often unbranched, (0–)2.5–5 mm long, thick, obscurely quadrangular to terete, green; secondary axils almost sessile when the peduncle is branched; flowers umbellate, 3–14 flowers per inflorescence; minute bracts at the base of the petiole early caducous, brown. ***Flowers*** pedicel 0.4–1 cm long, white; hypantho-calyx broadly infundibuliform to pyriform with an abrupt medial inflation, 1.3–1.5 mm long, 1.8–2.3 mm wide, outside smooth, white; calyx lobes 4, 0.4–0.5 mm long, 1.2–1.4 mm wide, obtuse to acute at apex; epigynous chamber smooth, without any furrows; exposed petals dome-shaped with an apiculate apex and 4 grooves radiating from the centre of each calyx lobe, white at anthesis, reflexed, 1.5–2 mm long, 1.2–1.6 mm wide; filaments 2.6–3.0 mm long, purple-blue; anther connective, curved, 1.2–1.3 mm long, 0.4–0.6 mm wide, purple-blue; with a prominent red gland; anthers pale brownish yellow; style 3.5–3.8 mm, grey. ***Fruits*** 1–5(–7) per inflorescence, reducing in number at maturity, on stiff pedicels up to 1–4 mm; depressed globose to oblate, 6–8 × 3–6 mm diameter, topped by a persistent calycinal crown; surface smooth, white at immature stage, indigo-blue at partial maturity, then turning to blackish purple at maturity; cotyledons hardly wrinkled.

**Figure 5.**
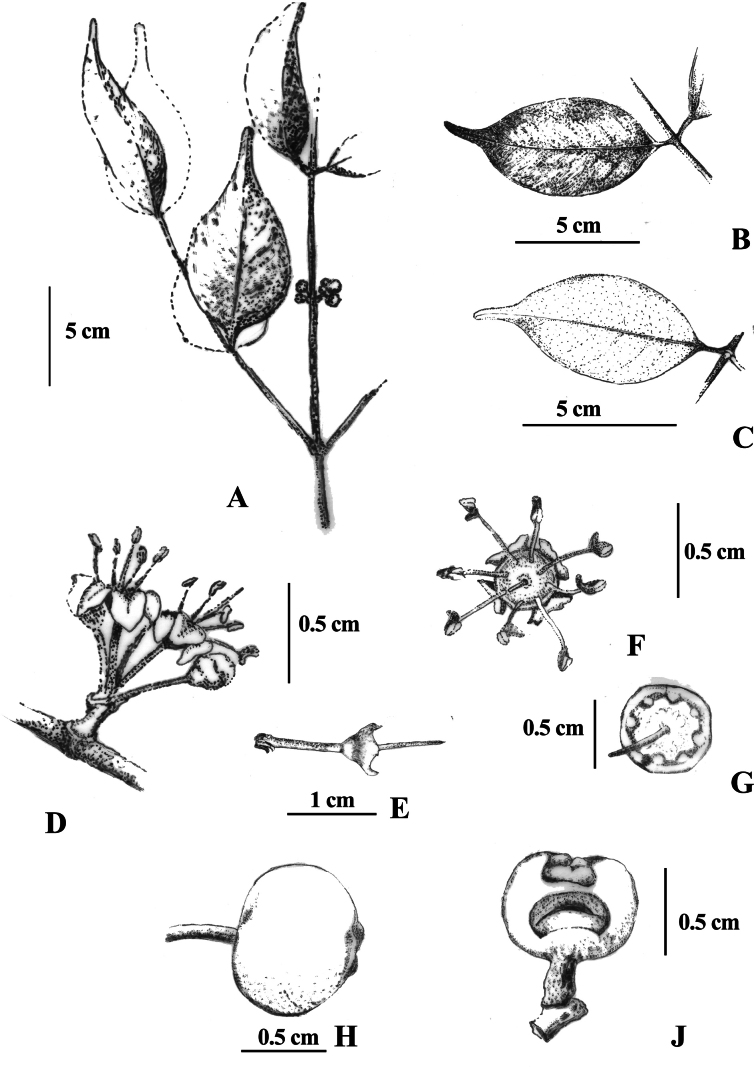
Line drawing of *Memecylonrostratum* Thwaites; **A.** Branch with immature fruits; **B.** Lamina upper side; **C.** Lamina under side; **D.** Dorsal view of flower; **E.** Lateral view of the flower just after anthesis; **F.** Dorsal view of the flower; **G.** Dorsal view of epigynous chamber after anthesis; **H.** Partially mature fruit; **J.** Longitudinal section of a partially mature fruit.

#### Distribution and habitat.

Southwestern lowland rainforests of Sri Lanka except the coastal zone, in the elevation range 200-–500 m (Fig. 3). PDA00002921! sheet contains information on three localities in Kandy District (the specimens were mounted by Thwaites in the 1800s). No further collections have been reported from natural habitats in the surroundings other than from the trees planted at the Royal Botanical Gardens, Peradeniya. It is thus possible that this historical gathering information may not relate to this species. *Memecylonrostratum* is usually confined to the upper level of the rainforest understorey, often on ridges within a given topography.

#### Phenology.

The main flowering season was reported from March to May, with fruits produced from May to August. A second season produces flowers from September to November, with fruits in December to February.

#### Notes.

Trimen (1894) mentioned that flowers of *Memecylonrostratum* are very pale blue, while Alston (1931), in his key to the species, repeated the same. The protologue mentioned that the petals were white. This was not necessarily a misconception: the petals are white while the inside of the hypanthocalyx and anther connectives are pale blue in this species. This is evident also from the drawing curated at PDA, which was based on syntypes.

Bremer (1979) lectotypified the name as ‘C.P. 1560 PDA’ while considering C.P. 1560 in BM, K & US as iso-lectotypes. We found that C.P. 1560 at PDA consisting of two herbarium sheets. PDA00002923! contains four branchlets with some detached leaves and carries no information about the gathering. PDA00002921! contains 6 branchlet fragments, one of which retains three attached leaves; the others lack leaves, though the detached leaves are pasted separately. The branchlet with the leaves has immature flower buds, while another branchlet that lacks leaves too, bears some young flower buds. An indistinct pencil notation on this sheet contains information on three gatherings (Hantana, Gardner; Deltota, in flower; Meda Mahanuwara, July 1852), though this cannot be explicitly assigned to the branchlets glued onto the sheet. In any event, because it is composed of multiple gatherings, Bremer’s lectotypification is incorrect. The present lectotype designation rectifies that deficiency and thereby stabilizes the identity of this species. As in the previous species, we refrain from considering other C.P. 1560 specimens as iso-lectotypes. Further, we have not included the C.P. 1560 specimens accessioned in herbaria outside Sri Lanka under the ‘specimens examined’, pending the availability of magnified images of the flowers, and since we have encountered similar looking but evidently undescribed species in the field.

Bremer (1979, 1987) quoted *Waas 509* (PDA, US) under this species, which is a species of *Eugenia* [probably *E.mooniana* Wight] (Myrtaceae). The specimens associated with C.P. 2684 and *NBK 315* quoted in these publications (Bremer 1979, 1987) belong to *M.elegantulum*. The specimens cited as *Waas & Peeris 551* and *Cramer 3720* do not exhibit sufficient detail to recognize them explicitly as *Memecylonrostratum*.

#### Specimens examined.

• **Sri Lanka: Kandy District**: Peradeniya, *n.d.*, *F.Fagerlind 4595* (E01411685; US2955736); • Royal Botanic Garden, Peradeniya, 10 iii 1979, *Kostermans 27421* (L.2545523, BR0000030741959, PDA[2 sheets]); • ibid., 25 v 1980, *Kostermans 28473* (L.2545522); • ibid., 13 ii 1904, C.C.Hosseus 12 (M0168532); • ibid., 2022 ix 16, *H.Jayasinghe HDJ 1693* (PDA); • ibid., 1964 v 26, *D.Amaratunga 818* (PDA); • ibid., 1955 v 30, *T.B.Worthington 6746* (PDA**Ratnapura District**: Sinharaja forest, between Heend Dola & Gallen Dola, 1989 iv 26, *A.H.M.Jayasuriya & S.Balasubramaniam 4697* (PDA); • Mulawella trail, Sinharaja, 2023 v 03, *H.Jayasinghe & D.Samarasinghe HDJ 2197* (PDA); Walankanda, 2 v 1976, *S.Waas 1557* (E01411686, L.2545521, PDA [2 sheets]) • **Kalutara District**: East Kalugala Forest, 1 v 1976, *S.Waas 1534* (E01411684, L.2545520, PDA [2 sheets]) • **Galle District**: Kanneliya forest near Hiniduma, 7 vi 1973, *Kostermans 24727* (L.2545524, US2955733); • ibid., 25 vii 1976, *A.H.M.Jayasuriya* & *A.J.Kostermans 2371* (P05255614, PDA); • Kalubowitiyana, 2023 xi 06, *H.Jayasinghe, D.Dhanushka, S.Kanishka & D.Samarasinghe HDJ 2557* (PDA); • Opatha, 2024 v 05, *H.Jayasinghe, D.Samarasinghe, A.Perera, P.Jayasundara HDJ 3061* (PDA).

## Supplementary Material

XML Treatment for Memecylon elegantulum

XML Treatment for Memecylon rostratum

## ﻿Additional information

### Author ORCIDs

Amila Perera https://orcid.org/0000-0002-7045-0790

Himesh Jayasinghe https://orcid.org/0000-0001-5308-9158

Bhathiya Gopallawa https://orcid.org/0000-0003-4293-7988

Isuru Madawala https://orcid.org/0000-0002-0462-9426

Nimal Gunatilleke https://orcid.org/0000-0003-3271-2945

Nalaka Geekiyanage https://orcid.org/0000-0002-7400-3453

### Data availability

All of the data that support the findings of this study are available in the main text.
